# Supplementary material for: Targeting NETosis in Acute Brain Injury: A Systematic Review of Preclinical and Clinical Evidence
Source: Cells. 2024 Sep 14;13(18):1553. doi: 10.3390/cells13181553 (PMC11440106; doi:10.3390/cells13181553)
Supplement: Supplementary file 1 [file cells-13-01553-s001.zip › cells-3171809-supplementary.pdf]

Supplementary materials

TABLE S1. Quality assessment of experimental animal studies

SYRCLE'S ROB	Random sequence generation	Baseline Characteristics	Allocation concealment	Random Housing	Blinding	Random Outcome Assessment	Blinding of outcome assessment	Incomplete outcome data	Selective reporting	Other bias
WU (2023)	X	+	X	?	X	?	?	X	?	+
DENORME (2022)	?	+	?	?	?	?	+	?	?	+
KIM (2020)	+	+	+	?	?	?	?	?	?	+
DE MEYER (2012)	?	+	?	?	?	?	+	+	+	+
KMET'OVA (2022)	?	+	?	+	?	X	+	+	+	+
WANG (2016)	?	+	?	?	?	?	?	?	?	+
SILLESEN (2013)	+	+	X	?	X	?	?	+	+	+

Comments: in Kmet'ova et al, outcome (functional outcome at 3 days) was assessed in all the survivors; Sillesen randomised 1:1 (randomisation performed but with allocation concealment issues).

TABLE S2. Quality assessment of non-randomised interventional human studies

ROBINS -I TOOL	Bias due to confounding	Bias in selection of participants into the study	Bias in classification of interventions	Bias due to deviations from intended interventions	Bias due to missing data	Bias in measurement of outcomes	Bias in selection of reported result	OVERALL JUDGEMENT
HUANG (2022)	X	?	X	+	+	?	X	X

Comments: high risk of bias in classification of interventions as conventional strategy (control group) was not clearly stated; possible bias in measurement of outcomes because it was not reported if outcome assessors were blind to intervention received. Besides, this study was a small population trial, including 30 patients affected by stroke and 15 healthy control individuals.

Legend

+	yes
?	unclear
X	no

FIGURE S1. Checklist for cohort studies on human populations

JBI CRITICAL APPRAISAL CHECKLIST FOR COHORT STUDIES

Reviewer_ _________SAVI M_____________________Date_________12/04/2024__________

Author_________GAO_________________________Year__2024_______ Record Number__1_______

	Yes	No	Unclear	Not applicable
1. Were the two groups similar and recruited from the same population?	☒	☐	☐	☐
2. Were the exposures measured similarly to assign people to both exposed and unexposed groups?	☒	☐	☐	☐
3. Was the exposure measured in a valid and reliable way?	☒	☐	☐	☐
4. Were confounding factors identified?	☒	☐	☐	☐
5. Were strategies to deal with confounding factors stated?	☐	☒	☐	☐
6. Were the groups/participants free of the outcome at the start of the study (or at the moment of exposure)?	☐	☐	☐	☒
7. Were the outcomes measured in a valid and reliable way?	☒	☐	☐	☐
8. Was the follow up time reported and sufficient to be long enough for outcomes to occur?	☒	☐	☐	☐
9. Was follow up complete, and if not, were the reasons to loss to follow up described and explored?	☒	☐	☐	☐
10. Were strategies to address incomplete follow up utilized?	☐	☐	☒	☐
11. Was appropriate statistical analysis used?	☒	☐	☐	☐

Overall appraisal: Include ☒ Exclude ☐ Seek further info ☐

Comments (Including reason for exclusion): possible bias for confounding factors (significant baseline difference in some risk factors (LDL, platelets count, WBC, D-Dimer, fibrinogen) in stroke population vs controls; no bias in selection of participants in the study; no bias due to missing data; no bias in the selection of reported results; no methodological issues

© JBI, 2020. All rights reserved. JBI grants use of these tools for research purposes only. All other enquiries should be sent to jbisynthesis@adelaide.edu.au.

JBI CRITICAL APPRAISAL CHECKLIST FOR COHORT STUDIES

Reviewer_ ________SAVI M____________________Date_______12/04/2024_________

Author________TIWARI___________________Year__2023_______ Record Number__2_______

	Yes	No	Unclear	Not applicable
1. Were the two groups similar and recruited from the same population?	☒	☐	☐	☐
2. Were the exposures measured similarly to assign people to both exposed and unexposed groups?	☐	☐	☐	☒
3. Was the exposure measured in a valid and reliable way?	☒	☐	☐	☐
4. Were confounding factors identified?	☐	☒	☐	☐
5. Were strategies to deal with confounding factors stated?	☐	☒	☐	☐
6. Were the groups/participants free of the outcome at the start of the study (or at the moment of exposure)?	☐	☐	☐	☒
7. Were the outcomes measured in a valid and reliable way?	☒	☐	☐	☐
8. Was the follow up time reported and sufficient to be long enough for outcomes to occur?	☒	☐	☐	☐
9. Was follow up complete, and if not, were the reasons to loss to follow up described and explored?	☒	☐	☐	☐
10. Were strategies to address incomplete follow up utilized?	☐	☐	☒	☐
11. Was appropriate statistical analysis used?	☒	☐	☐	☐

Overall appraisal: Include ☒ Exclude ☐ Seek further info ☐

Comments (Including reason for exclusion): possible bias due to confounding factors, as neither the n° of control subjects nor their baseline characteristics were reported; possible bias in selection of participants in the study; bias due to missing data (control); possible bias in the selection of reported results; concern on reliability of Vajpeeyee 2020 and Tiwari 2023 as both come from the same department and established a threshold of 10.000 kilogenom eq/L of cfDNA to be associated with a higher probability of poor functional outcome at 3 months in AIS; no methodological issues.

© JBI, 2020. All rights reserved. JBI grants use of these tools for research purposes only. All other enquiries should be sent to jbisynthesis@adelaide.edu.au.

JBI CRITICAL APPRAISAL CHECKLIST FOR COHORT STUDIES

Reviewer_ _________SAVI M____________________Date________12/04/2024_________

Author_______ZHANG_________________________Year__2023_______ Record Number__3_______

		Yes	No	Unclear	Not applicable
1.	Were the two groups similar and recruited from the same population?	☒	☐	☐	☐
2.	Were the exposures measured similarly to assign people to both exposed and unexposed groups?	☐	☐	☐	☒
3.	Was the exposure measured in a valid and reliable way?	☒	☐	☐	☐
4.	Were confounding factors identified?	☒	☐	☐	☐
5.	Were strategies to deal with confounding factors stated?	☒	☐	☐	☐
6.	Were the groups/participants free of the outcome at the start of the study (or at the moment of exposure)?	☐	☐	☒	☐
7.	Were the outcomes measured in a valid and reliable way?	☒	☐	☐	☐
8.	Was the follow up time reported and sufficient to be long enough for outcomes to occur?	☒	☐	☐	☐
9.	Was follow up complete, and if not, were the reasons to loss to follow up described and explored?	☒	☐	☐	☐
10.	Were strategies to address incomplete follow up utilized?	☐	☐	☐	☒
11.	Was appropriate statistical analysis used?	☒	☐	☐	☐

Overall appraisal: Include ☒ Exclude ☐ Seek further info ☐

Comments (Including reason for exclusion): confounding factors were appropriately detected and addressed by adjusted univariate analysis; no bias in selection of participants in the study; no bias due to missing data; no bias in the selection of reported results; no methodological issues

© JBI, 2020. All rights reserved. JBI grants use of these tools for research purposes only. All other enquiries should be sent to jbisynthesis@adelaide.edu.au.

JBI CRITICAL APPRAISAL CHECKLIST FOR COHORT STUDIES

Reviewer_ _________SAVI M_____________________Date________12/04/2024__________

Author________DENORME_____________________Year__2022_______ Record Number__4______

		Yes	No	Unclear	Not applicable
1.	Were the two groups similar and recruited from the same population?	☒	☐	☐	☐
2.	Were the exposures measured similarly to assign people to both exposed and unexposed groups?	☒	☐	☐	☐
3.	Was the exposure measured in a valid and reliable way?	☒	☐	☐	☐
4.	Were confounding factors identified?	☐	☒	☐	☐
5.	Were strategies to deal with confounding factors stated?	☐	☒	☐	☐
6.	Were the groups/participants free of the outcome at the start of the study (or at the moment of exposure)?	☐	☐	☒	☐
7.	Were the outcomes measured in a valid and reliable way?	☒	☐	☐	☐
8.	Was the follow up time reported and sufficient to be long enough for outcomes to occur?	☒	☐	☐	☐
9.	Was follow up complete, and if not, were the reasons to loss to follow up described and explored?	☒	☐	☐	☐
10.	Were strategies to address incomplete follow up utilized?	☐	☐	☐	☒
11.	Was appropriate statistical analysis used?	☒	☐	☐	☐

Overall appraisal: Include ☒ Exclude ☐ Seek further info ☐

Comments (Including reason for exclusion): possible bias for confounding factors (significant baseline difference in risk factors such as hyperlipidemia and hypertension in stroke population vs controls, not statistically significant but noticeable age difference between the two arms; no bias in selection of participants in the study; no bias due to missing data; no bias in the selection of reported results; no methodological issues

© JBI, 2020. All rights reserved. JBI grants use of these tools for research purposes only. All other enquiries should be sent to jbisynthesis@adelaide.edu.au.

JBI CRITICAL APPRAISAL CHECKLIST FOR COHORT STUDIES

Reviewer_ _________SAVI M_____________________Date________12/04/2024__________

Author_________GROSSE______________________Year__2022_______ Record Number__5_______

		Yes	No	Unclear	Not applicable
1.	Were the two groups similar and recruited from the same population?	☒	☐	☐	☐
2.	Were the exposures measured similarly to assign people to both exposed and unexposed groups?	☒	☐	☐	☐
3.	Was the exposure measured in a valid and reliable way?	☒	☐	☐	☐
4.	Were confounding factors identified?	☒	☐	☐	☐
5.	Were strategies to deal with confounding factors stated?	☒	☐	☐	☐
6.	Were the groups/participants free of the outcome at the start of the study (or at the moment of exposure)?	☐	☐	☒	☐
7.	Were the outcomes measured in a valid and reliable way?	☒	☐	☐	☐
8.	Was the follow up time reported and sufficient to be long enough for outcomes to occur?	☒	☐	☐	☐
9.	Was follow up complete, and if not, were the reasons to loss to follow up described and explored?	☒	☐	☐	☐
10.	Were strategies to address incomplete follow up utilized?	☐	☐	☒	☐
11.	Was appropriate statistical analysis used?	☒	☐	☐	☐

Overall appraisal: Include ☒ Exclude ☐ Seek further info ☐

Comments (Including reason for exclusion): confounding factors were appropriately detected and addressed by regression analysis; no bias in selection of participants in the study; no bias due to missing data; no bias in the selection of reported results; no methodological issues

© JBI, 2020. All rights reserved. JBI grants use of these tools for research purposes only. All other enquiries should be sent to jbisynthesis@adelaide.edu.au.

JBI CRITICAL APPRAISAL CHECKLIST FOR COHORT STUDIES

Reviewer_ _________SAVI M____________________Date________12/04/2024_________

Author_________CUI_______________________Year__2020_______ Record Number__6______

	Yes	No	Unclear	Not applicable
1. Were the two groups similar and recruited from the same population?	☐	☒	☐	☐
2. Were the exposures measured similarly to assign people to both exposed and unexposed groups?	☐	☐	☐	☒
3. Was the exposure measured in a valid and reliable way?	☐	☒	☐	☐
4. Were confounding factors identified?	☐	☒	☐	☐
5. Were strategies to deal with confounding factors stated?	☐	☒	☐	☐
6. Were the groups/participants free of the outcome at the start of the study (or at the moment of exposure)?	☐	☐	☐	☐
7. Were the outcomes measured in a valid and reliable way?	☒	☐	☐	☐
8. Was the follow up time reported and sufficient to be long enough for outcomes to occur?	☒	☐	☐	☐
9. Was follow up complete, and if not, were the reasons to loss to follow up described and explored?	☐	☒	☐	☐
10. Were strategies to address incomplete follow up utilized?	☐	☒	☐	☐
11. Was appropriate statistical analysis used?	☒	☐	☐	☐

Overall appraisal: Include ☒ Exclude ☐ Seek further info ☐

Comments (Including reason for exclusion): baseline characteristics of population not reported; possible bias for confounding factors, possible bias in selection of participants in the study; possible bias due to missing data; possible bias in the selection of reported results; methodological issues as definition/diagnosis of disease (exposure) is missing

© JBI, 2020. All rights reserved. JBI grants use of these tools for research purposes only. All other enquiries should be sent to jbisynthesis@adelaide.edu.au.

JBI CRITICAL APPRAISAL CHECKLIST FOR COHORT STUDIES

Reviewer_ ________SAVI M____________________Date_______12/04/2024_________

Author________LIM_______________________Year__2020_______ Record Number__7______

		Yes	No	Unclear	Not applicable
1.	Were the two groups similar and recruited from the same population?	☒	☐	☐	☐
2.	Were the exposures measured similarly to assign people to both exposed and unexposed groups?	☐	☐	☐	☒
3.	Was the exposure measured in a valid and reliable way?	☒	☐	☐	☐
4.	Were confounding factors identified?	☐	☒	☐	☐
5.	Were strategies to deal with confounding factors stated?	☐	☒	☐	☐
6.	Were the groups/participants free of the outcome at the start of the study (or at the moment of exposure)?	☐	☐	☐	☒
7.	Were the outcomes measured in a valid and reliable way?	☒	☐	☐	☐
8.	Was the follow up time reported and sufficient to be long enough for outcomes to occur?	☒	☐	☐	☐
9.	Was follow up complete, and if not, were the reasons to loss to follow up described and explored?	☒	☐	☐	☐
10.	Were strategies to address incomplete follow up utilized?	☐	☒	☐	☐
11.	Was appropriate statistical analysis used?	☒	☐	☐	☐

Overall appraisal: Include ☒ Exclude ☐ Seek further info ☐

Comments (Including reason for exclusion): no significant baseline demographical differences between stroke patients and healthy control; possible bias for confounding factors; no bias in selection of participants in the study; no bias due to missing data; no bias in the selection of reported results; no methodological issues

© JBI, 2020. All rights reserved. JBI grants use of these tools for research purposes only. All other enquiries should be sent to jbisynthesis@adelaide.edu.au.

JBI CRITICAL APPRAISAL CHECKLIST FOR COHORT STUDIES

Reviewer_ _________SAVI M____________________Date________12/04/2024__________

Author_________ VAJPEEYEE_____________________Year__2020____ Record Number__8____

	Question	Yes	No	Unclear	Not applicable
1.	Were the two groups similar and recruited from the same population?	☐	☐	☐	☒
2.	Were the exposures measured similarly to assign people to both exposed and unexposed groups?	☐	☐	☐	☒
3.	Was the exposure measured in a valid and reliable way?	☒	☐	☐	☐
4.	Were confounding factors identified?	☐	☒	☐	☐
5.	Were strategies to deal with confounding factors stated?	☐	☒	☐	☐
6.	Were the groups/participants free of the outcome at the start of the study (or at the moment of exposure)?	☐	☐	☐	☒
7.	Were the outcomes measured in a valid and reliable way?	☒	☐	☐	☐
8.	Was the follow up time reported and sufficient to be long enough for outcomes to occur?	☒	☐	☐	☐
9.	Was follow up complete, and if not, were the reasons to loss to follow up described and explored?	☒	☐	☐	☐
10.	Were strategies to address incomplete follow up utilized?	☐	☒	☐	☐
11.	Was appropriate statistical analysis used?	☒	☐	☐	☐

Overall appraisal: Include ☒ Exclude ☐ Seek further info ☐

Comments (Including reason for exclusion): possible bias due to confounding factors; no bias in selection of participants in the study; concern on reliability of Vajpeeyee 2020 and Tiwari 2023 as both studies come from the same department and established a threshold of 10.000 kilogenom eq/L of cfDNA to be associated with a higher probability of poor functional outcome at 3 months in AIS; no methodological issues

© JBI, 2020. All rights reserved. JBI grants use of these tools for research purposes only. All other enquiries should be sent to jbisynthesis@adelaide.edu.au.

JBI CRITICAL APPRAISAL CHECKLIST FOR COHORT STUDIES

Reviewer_ _________SAVI M____________________Date________12/04/2024_________

Author_________VALLES_____________________Year__2020____ Record Number__9_____

	Yes	No	Unclear	Not applicable
12. Were the two groups similar and recruited from the same population?	☐	☐	☒	☐
13. Were the exposures measured similarly to assign people to both exposed and unexposed groups?	☐	☐	☐	☒
14. Was the exposure measured in a valid and reliable way?	☒	☐	☐	☐
15. Were confounding factors identified?	☐	☒	☐	☐
16. Were strategies to deal with confounding factors stated?	☐	☒	☐	☐
17. Were the groups/participants free of the outcome at the start of the study (or at the moment of exposure)?	☐	☐	☐	☒
18. Were the outcomes measured in a valid and reliable way?	☒	☐	☐	☐
19. Was the follow up time reported and sufficient to be long enough for outcomes to occur?	☒	☐	☐	☐
20. Was follow up complete, and if not, were the reasons to loss to follow up described and explored?	☒	☐	☐	☐
21. Were strategies to address incomplete follow up utilized?	☐	☒	☐	☐
22. Was appropriate statistical analysis used?	☒	☐	☐	☐

Overall appraisal: Include ☒ Exclude ☐ Seek further info ☐

Comments (Including reason for exclusion): possible bias due to confounding factors as baseline characteristics of control subjects were not reported; possible bias in selection of participants in the study; possible bias due to missing data and in the selection of reported results in follow-up; no methodological issues

© JBI, 2020. All rights reserved. JBI grants use of these tools for research purposes only. All other enquiries should be sent to jbisynthesis@adelaide.edu.au.

JBI CRITICAL APPRAISAL CHECKLIST FOR COHORT STUDIES

Reviewer_ _________SAVI M_____________________Date________12/04/2024_________

Author_________CAO_________________________Year__2023_______ Record Number__10_____

	Yes	No	Unclear	Not applicable
1. Were the two groups similar and recruited from the same population?	☐	☒	☐	☐
2. Were the exposures measured similarly to assign people to both exposed and unexposed groups?	☐	☐	☐	☒
3. Was the exposure measured in a valid and reliable way?	☒	☐	☐	☐
4. Were confounding factors identified?	☐	☒	☐	☐
5. Were strategies to deal with confounding factors stated?	☐	☒	☐	☐
6. Were the groups/participants free of the outcome at the start of the study (or at the moment of exposure)?	☐	☐	☐	☒
7. Were the outcomes measured in a valid and reliable way?	☒	☐	☐	☐
8. Was the follow up time reported and sufficient to be long enough for outcomes to occur?	☒	☐	☐	☐
9. Was follow up complete, and if not, were the reasons to loss to follow up described and explored?	☒	☐	☐	☐
10. Were strategies to address incomplete follow up utilized?	☐	☒	☐	☐
11. Was appropriate statistical analysis used?	☒	☐	☐	☐

Overall appraisal: Include ☒ Exclude ☐ Seek further info ☐

Comments (Including reason for exclusion): possible bias due to confounding factors and selection of participants as baseline characteristics of head trauma patients and control subjects were not reported; possible bias due to missing data and in the selection of reported results in follow-up; no methodological issues

© JBI, 2020. All rights reserved. JBI grants use of these tools for research purposes only. All other enquiries should be sent to jbisynthesis@adelaide.edu.au.

JBI CRITICAL APPRAISAL CHECKLIST FOR COHORT STUDIES

Reviewer_ _________SAVI M____________________Date________12/04/2024__________

Author_________MI___________________Year__2023_______ Record Number__11_______

		Yes	No	Unclear	Not applicable
1.	Were the two groups similar and recruited from the same population?	☐	☒	☐	☐
2.	Were the exposures measured similarly to assign people to both exposed and unexposed groups?	☐	☐	☐	☒
3.	Was the exposure measured in a valid and reliable way?	☐	☒	☐	☐
4.	Were confounding factors identified?	☐	☒	☐	☐
5.	Were strategies to deal with confounding factors stated?	☐	☒	☐	☐
6.	Were the groups/participants free of the outcome at the start of the study (or at the moment of exposure)?	☐	☐	☐	☒
7.	Were the outcomes measured in a valid and reliable way?	☒	☐	☐	☐
8.	Was the follow up time reported and sufficient to be long enough for outcomes to occur?	☒	☐	☐	☐
9.	Was follow up complete, and if not, were the reasons to loss to follow up described and explored?	☒	☐	☐	☐
10.	Were strategies to address incomplete follow up utilized?	☐	☒	☐	☐
11.	Was appropriate statistical analysis used?	☒	☐	☐	☐

Overall appraisal: Include ☒ Exclude ☐ Seek further info ☐

Comments (Including reason for exclusion): possible bias due to confounding factors and selection of participants, as baseline characteristics of patients and control subjects were not reported; bias due to missing data; possible bias in the selection of reported results; no methodological issues

© JBI, 2020. All rights reserved. JBI grants use of these tools for research purposes only. All other enquiries should be sent to jbisynthesis@adelaide.edu.au.

JBI CRITICAL APPRAISAL CHECKLIST FOR COHORT STUDIES

Reviewer_ ________SAVI M____________________Date_______12/04/2024_________

Author________BEN ZVI____________________Year__2022_______ Record Number__12______

	Yes	No	Unclear	Not applicable
1. Were the two groups similar and recruited from the same population?	☐	☐	☐	☒
2. Were the exposures measured similarly to assign people to both exposed and unexposed groups?	☐	☐	☐	☒
3. Was the exposure measured in a valid and reliable way?	☒	☐	☐	☐
4. Were confounding factors identified?	☒	☐	☐	☐
5. Were strategies to deal with confounding factors stated?	☒	☐	☐	☐
6. Were the groups/participants free of the outcome at the start of the study (or at the moment of exposure)?	☐	☐	☐	☒
7. Were the outcomes measured in a valid and reliable way?	☒	☐	☐	☐
8. Was the follow up time reported and sufficient to be long enough for outcomes to occur?	☒	☐	☐	☐
9. Was follow up complete, and if not, were the reasons to loss to follow up described and explored?	☒	☐	☐	☐
10. Were strategies to address incomplete follow up utilized?	☐	☒	☐	☐
11. Was appropriate statistical analysis used?	☒	☐	☐	☐

Overall appraisal: Include ☒ Exclude ☐ Seek further info ☐

Comments (Including reason for exclusion): possible bias due to selection of participants (even if per inclusion criteria only adult patients under 67 years old should have been recruited, three older patients were enrolled); possible bias due to missing data and in the selection of reported results in follow-up; no methodological issues

© JBI, 2020. All rights reserved. JBI grants use of these tools for research purposes only. All other enquiries should be sent to jbisynthesis@adelaide.edu.au.

JBI CRITICAL APPRAISAL CHECKLIST FOR COHORT STUDIES

Reviewer_ ________SAVI M____________________Date_______12/04/2024_________

Author________ HAZELDINE_________________Year__2021______ Record Number__13______

	Yes	No	Unclear	Not applicable
1. Were the two groups similar and recruited from the same population?	☐	☒	☐	☐
2. Were the exposures measured similarly to assign people to both exposed and unexposed groups?	☒	☐	☐	☐
3. Was the exposure measured in a valid and reliable way?	☒	☐	☐	☐
4. Were confounding factors identified?	☐	☒	☐	☐
5. Were strategies to deal with confounding factors stated?	☐	☒	☐	☐
6. Were the groups/participants free of the outcome at the start of the study (or at the moment of exposure)?	☐	☐	☐	☒
7. Were the outcomes measured in a valid and reliable way?	☒	☐	☐	☐
8. Was the follow up time reported and sufficient to be long enough for outcomes to occur?	☒	☐	☐	☐
9. Was follow up complete, and if not, were the reasons to loss to follow up described and explored?	☐	☒	☐	☐
10. Were strategies to address incomplete follow up utilized?	☐	☒	☐	☐
11. Was appropriate statistical analysis used?	☒	☐	☐	☐

Overall appraisal: Include ☒ Exclude ☐ Seek further info ☐

Comments (Including reason for exclusion): possible bias for confounding factors (baseline difference in sex between control patients and trauma population); no bias in selection of participants in the study; possible bias due to missing data as patients were lost during data collection; possible bias in the selection of reported results; no methodological issues

© JBI, 2020. All rights reserved. JBI grants use of these tools for research purposes only. All other enquiries should be sent to jbisynthesis@adelaide.edu.au.

JBI CRITICAL APPRAISAL CHECKLIST FOR COHORT STUDIES

Reviewer_ _________SAVI M____________________Date________12/04/2024__________

Author_________ MARCATTI ___________________Year__2021_______ Record Number__14_____

	Yes	No	Unclear	Not applicable
1. Were the two groups similar and recruited from the same population?	☐	☒	☐	☐
2. Were the exposures measured similarly to assign people to both exposed and unexposed groups?	☐	☐	☐	☒
3. Was the exposure measured in a valid and reliable way?	☒	☐	☐	☐
4. Were confounding factors identified?	☐	☒	☐	☐
5. Were strategies to deal with confounding factors stated?	☐	☒	☐	☐
6. Were the groups/participants free of the outcome at the start of the study (or at the moment of exposure)?	☐	☐	☐	☒
7. Were the outcomes measured in a valid and reliable way?	☒	☐	☐	☐
8. Was the follow up time reported and sufficient to be long enough for outcomes to occur?	☒	☐	☐	☐
9. Was follow up complete, and if not, were the reasons to loss to follow up described and explored?	☒	☐	☐	☐
10. Were strategies to address incomplete follow up utilized?	☐	☒	☐	☐
11. Was appropriate statistical analysis used?	☒	☐	☐	☐

Overall appraisal: Include ☒ Exclude ☐ Seek further info ☐

Comments (Including reason for exclusion): possible bias for confounding factors and in selection of participants, as baseline characteristics of both trauma patients and control subjects; no bias in the selection of reported results or due to missing data; no methodological issues

© JBI, 2020. All rights reserved. JBI grants use of these tools for research purposes only. All other enquiries should be sent to jbisynthesis@adelaide.edu.au.

JBI CRITICAL APPRAISAL CHECKLIST FOR COHORT STUDIES

Reviewer_ _________SAVI M____________________Date________12/04/2024__________

Author_________SHAKED_____________________Year__2021_______ Record Number__15_____

	Yes	No	Unclear	Not applicable
1. Were the two groups similar and recruited from the same population?	☒	☐	☐	☐
2. Were the exposures measured similarly to assign people to both exposed and unexposed groups?	☒	☐	☐	☐
3. Was the exposure measured in a valid and reliable way?	☒	☐	☐	☐
4. Were confounding factors identified?	☐	☒	☐	☐
5. Were strategies to deal with confounding factors stated?	☐	☒	☐	☐
6. Were the groups/participants free of the outcome at the start of the study (or at the moment of exposure)?	☐	☐	☐	☒
7. Were the outcomes measured in a valid and reliable way?	☒	☐	☐	☐
8. Was the follow up time reported and sufficient to be long enough for outcomes to occur?	☒	☐	☐	☐
9. Was follow up complete, and if not, were the reasons to loss to follow up described and explored?	☐	☐	☒	☐
10. Were strategies to address incomplete follow up utilized?	☐	☒	☐	☐
11. Was appropriate statistical analysis used?	☒	☐	☐	☐

Overall appraisal: Include ☒ Exclude ☐ Seek further info ☐

Comments (Including reason for exclusion: possible bias for confounding factors, as they were not addressed, and possible bias in selection of participants as baseline control characteristics were not reported; possible bias due to missing data as patients were lost during data collection; possible bias in the selection of reported results; no methodological issues

© JBI, 2020. All rights reserved. JBI grants use of these tools for research purposes only. All other enquiries should be sent to jbisynthesis@adelaide.edu.au.

JBI CRITICAL APPRAISAL CHECKLIST FOR COHORT STUDIES

Reviewer_ _________SAVI M____________________Date________12/04/2024_________

Author_______MORAES RODRIGUEZ FILHO________ Year__2014_______ Record Number__16_______

	Yes	No	Unclear	Not applicable
1. Were the two groups similar and recruited from the same population?	☒	☐	☐	☐
2. Were the exposures measured similarly to assign people to both exposed and unexposed groups?	☒	☐	☐	☐
3. Was the exposure measured in a valid and reliable way?	☒	☐	☐	☐
4. Were confounding factors identified?	☒	☐	☐	☐
5. Were strategies to deal with confounding factors stated?	☒	☐	☐	☐
6. Were the groups/participants free of the outcome at the start of the study (or at the moment of exposure)?	☐	☐	☐	☒
7. Were the outcomes measured in a valid and reliable way?	☒	☐	☐	☐
8. Was the follow up time reported and sufficient to be long enough for outcomes to occur?	☒	☐	☐	☐
9. Was follow up complete, and if not, were the reasons to loss to follow up described and explored?	☒	☐	☐	☐
10. Were strategies to address incomplete follow up utilized?	☐	☒	☐	☐
11. Was appropriate statistical analysis used?	☒	☐	☐	☐

Overall appraisal: Include ☒ Exclude ☐ Seek further info ☐

Comments (Including reason for exclusion): possible bias for confounding factors (difference in sex between control subjects and trauma patients); no bias in selection of participants; logistic regression was adjusted for confounders between survivors vs non-survivors; no bias in the selection of reported results; no methodological issues

© JBI, 2020. All rights reserved. JBI grants use of these tools for research purposes only. All other enquiries should be sent to jbisynthesis@adelaide.edu.au.

JBI CRITICAL APPRAISAL CHECKLIST FOR COHORT STUDIES

Reviewer_ _________SAVI M_____________________Date________12/04/2024__________

Author________MACHER______________________Year__2012_______ Record Number__17_______

		Yes	No	Unclear	Not applicable
1.	Were the two groups similar and recruited from the same population?	☒	☐	☐	☐
2.	Were the exposures measured similarly to assign people to both exposed and unexposed groups?	☒	☐	☐	☐
3.	Was the exposure measured in a valid and reliable way?	☒	☐	☐	☐
4.	Were confounding factors identified?	☐	☒	☐	☐
5.	Were strategies to deal with confounding factors stated?	☐	☒	☐	☐
6.	Were the groups/participants free of the outcome at the start of the study (or at the moment of exposure)?	☒	☐	☐	☐
7.	Were the outcomes measured in a valid and reliable way?	☒	☐	☐	☐
8.	Was the follow up time reported and sufficient to be long enough for outcomes to occur?	☒	☐	☐	☐
9.	Was follow up complete, and if not, were the reasons to loss to follow up described and explored?	☒	☐	☐	☐
10.	Were strategies to address incomplete follow up utilized?	☐	☒	☐	☐
11.	Was appropriate statistical analysis used?	☒	☐	☐	☐

Overall appraisal: Include ☒ Exclude ☐ Seek further info ☐

Comments (Including reason for exclusion): possible bias for confounding factors as logistic regression was not adjusted for possible confounders between survivors vs non-survivors; no bias in selection of participants; no bias in the selection of reported results; no bias due to missing data; no methodological issues

© JBI, 2020. All rights reserved. JBI grants use of these tools for research purposes only. All other enquiries should be sent to jbisynthesis@adelaide.edu.au.

JBI CRITICAL APPRAISAL CHECKLIST FOR COHORT STUDIES

Reviewer_ _________SAVI M_____________________Date________12/04/2024__________

Author______CAMPELLO YURGEL________________Year__2007______ Record Number__18______

		Yes	No	Unclear	Not applicable
1.	Were the two groups similar and recruited from the same population?	☒	☐	☐	☐
2.	Were the exposures measured similarly to assign people to both exposed and unexposed groups?	☒	☐	☐	☐
3.	Was the exposure measured in a valid and reliable way?	☒	☐	☐	☐
4.	Were confounding factors identified?	☐	☒	☐	☐
5.	Were strategies to deal with confounding factors stated?	☐	☒	☐	☐
6.	Were the groups/participants free of the outcome at the start of the study (or at the moment of exposure)?	☐	☐	☐	☒
7.	Were the outcomes measured in a valid and reliable way?	☒	☐	☐	☐
8.	Was the follow up time reported and sufficient to be long enough for outcomes to occur?	☒	☐	☐	☐
9.	Was follow up complete, and if not, were the reasons to loss to follow up described and explored?	☒	☐	☐	☐
10.	Were strategies to address incomplete follow up utilized?	☐	☒	☐	☐
11.	Was appropriate statistical analysis used?	☒	☐	☐	☐

Overall appraisal: Include ☒ Exclude ☐ Seek further info ☐

Comments (Including reason for exclusion): possible bias for confounding factors; no bias in selection of participants; no bias due to missing data; no bias in the selection of reported results; no methodological issues

© JBI, 2020. All rights reserved. JBI grants use of these tools for research purposes only. All other enquiries should be sent to jbisynthesis@adelaide.edu.au.

JBI CRITICAL APPRAISAL CHECKLIST FOR COHORT STUDIES

Reviewer_ ________SAVI M____________________Date_______12/04/2024_________

Author______WITSCH _______________Year__2022______ Record Number__19______

		Yes	No	Unclear	Not applicable
1.	Were the two groups similar and recruited from the same population?	☒	☐	☐	☐
2.	Were the exposures measured similarly to assign people to both exposed and unexposed groups?	☒	☐	☐	☐
3.	Was the exposure measured in a valid and reliable way?	☒	☐	☐	☐
4.	Were confounding factors identified?	☒	☐	☐	☐
5.	Were strategies to deal with confounding factors stated?	☐	☒	☐	☐
6.	Were the groups/participants free of the outcome at the start of the study (or at the moment of exposure)?	☒	☐	☐	☐
7.	Were the outcomes measured in a valid and reliable way?	☒	☐	☐	☐
8.	Was the follow up time reported and sufficient to be long enough for outcomes to occur?	☒	☐	☐	☐
9.	Was follow up complete, and if not, were the reasons to loss to follow up described and explored?	☒	☐	☐	☐
10.	Were strategies to address incomplete follow up utilized?	☐	☒	☐	☐
11.	Was appropriate statistical analysis used?	☒	☐	☐	☐

Overall appraisal: Include ☒ Exclude ☐ Seek further info ☐

Comments (Including reason for exclusion): DCI considered as the exposure factor, NET markers as outcome; possible bias due to confounding factors; no bias due to selection of participants; no bias due to missing data; no bias in the selection of reported results; no methodological issues

© JBI, 2020. All rights reserved. JBI grants use of these tools for research purposes only. All other enquiries should be sent to jbisynthesis@adelaide.edu.au.

JBI CRITICAL APPRAISAL CHECKLIST FOR COHORT STUDIES

Reviewer_ ________SAVI M____________________Date_______12/04/2024_________

Author______ZENG______________Year__2021______ Record Number__20______

		Yes	No	Unclear	Not applicable
1.	Were the two groups similar and recruited from the same population?	☐	☐	☒	☐
2.	Were the exposures measured similarly to assign people to both exposed and unexposed groups?	☐	☐	☐	☒
3.	Was the exposure measured in a valid and reliable way?	☐	☐	☒	☐
4.	Were confounding factors identified?	☐	☒	☐	☐
5.	Were strategies to deal with confounding factors stated?	☐	☒	☐	☐
6.	Were the groups/participants free of the outcome at the start of the study (or at the moment of exposure)?	☐	☐	☐	☒
7.	Were the outcomes measured in a valid and reliable way?	☒	☐	☐	☐
8.	Was the follow up time reported and sufficient to be long enough for outcomes to occur?	☒	☐	☐	☐
9.	Was follow up complete, and if not, were the reasons to loss to follow up described and explored?	☒	☐	☐	☐
10.	Were strategies to address incomplete follow up utilized?	☐	☒	☐	☐
11.	Was appropriate statistical analysis used?	☒	☐	☐	☐

Overall appraisal: Include ☒ Exclude ☐ Seek further info ☐

Comments (Including reason for exclusion): possible bias due to confounding factors and selection of participants, as baseline characteristics of patients and control subjects were not reported; bias due to missing data; possible bias in the selection of reported results; no methodological issues.

© JBI, 2020. All rights reserved. JBI grants use of these tools for research purposes only. All other enquiries should be sent to jbisynthesis@adelaide.edu.au.
